# Dietary Supplementation With *Bacillus subtilis* Direct-Fed Microbials Alters Chicken Intestinal Metabolite Levels

**DOI:** 10.3389/fvets.2020.00123

**Published:** 2020-03-04

**Authors:** Inkyung Park, Noah P. Zimmerman, Alexandra H. Smith, Thomas G. Rehberger, Erik. P. Lillehoj, Hyun S. Lillehoj

**Affiliations:** ^1^Animal Bioscience and Biotechnology Laboratory, United States Department of Agriculture, Beltsville Agricultural Research Center, Agricultural Research Service, Beltsville, MD, United States; ^2^Arm & Hammer Animal and Food Production, Waukesha, WI, United States; ^3^Department of Pediatrics, University of Maryland School of Medicine, Baltimore, MD, United States

**Keywords:** amino acid, fatty acid, gut, metabolomics, nucleoside, probiotics

## Abstract

Direct-fed microbials (DFMs) are dietary supplements containing live microorganisms which confer a performance and health benefit to the host, but the mechanisms are unclear. Here, a metabolomics approach was used to identify changes in intestinal metabolite levels in chickens fed an unsupplemented diet or a diet supplemented with *B. subtilis* strain 1781 or strain 747. Body weight gains of chickens fed the *B. subtilis*-supplemented diets were increased up to 5.6% in the *B. subtilis* 1781 group and 7.6% in the *B. subtilis* 747 group compared with chickens fed the unsupplemented diet. Compared with unsupplemented controls, the levels of 83 metabolites were altered (*p* < 0.05) (25 increased, 58 decreased) in chickens given the *B. subtilis* 1781-supplemented diet, while 50 were altered (*p* < 0.05) (12 increased, 38 decreased) with the *B. subtilis* 747-supplemented diet. Twenty-two metabolites were altered (*p* < 0.05) (18 increased, 4 decreased) in the *B. subtilis* 1781 vs. *B. subtilis* 747 groups. A random forest analysis of the *B. subtilis* 1781 vs. control groups gave a predictive accuracy of 87.5%, while that of the *B. subtilis* 747 vs. control groups was 62.5%. A random forest analysis of the *B. subtilis* 1781 vs. *B. subtilis* 747 groups gave a predictive accuracy of 75.0%. Changes in the levels of these intestinal biochemicals provided a distinctive biochemical signature unique to each *B. subtilis*-supplemented group, and were characterized by alterations in the levels of dipeptides (alanylleucine, glutaminylleucine, phenylalanylalanine, valylglutamine), nucleosides (*N*1-methyladenosine, *N*6-methyladenosine, guanine, 2-deoxyguanosine), fatty acids (sebacate, valerylglycine, linoleoylcholine), and carbohydrates (fructose). These results provide the foundation for future studies to identify biochemicals that might be used to improve poultry growth performance in the absence of antibiotic growth promoters.

## Introduction

Microbial resistance to antibiotics is a serious and growing public health problem worldwide with multiple causes (1, 2). One likely contributory factor is the widespread use of antibiotics as growth promoters in commercial livestock and poultry production. Dietary antibiotic growth promoters (AGPs) have been used in the food animal industry for more than 60 years to increase feed efficiency and improve growth performance (3, 4). Increasing evidence, however, suggests that AGP use in food animal production leads to the development of antibiotic resistance among the endogenous gut commensal microbiota with the potential for transfer to the human population (5–9). Consequently, there is an unmet need to elucidate the molecular and cellular interactions between the intestinal microbiota and host that might be modulated by other means to promote food animal growth in the absence of AGPs. Among the substances that have been tested as alternatives to AGPs are probiotics and direct-fed microbials (DFMs) (10–12).

DFMs are viable, naturally occurring microbial cultures, including bacteria belonging to the genera *Lactobacillus, Enterococcus, Streptococcus, Bifidobacterium*, and *Propionibacterium*, which when fed to the host animal, generate a beneficial health response through their ability to modulate the diversity and composition of the gut microbiota. Various species of *Bacillus* also have been tested as DFMs on the basis of their ability to inhibit pathogens by producing antimicrobials (13–15). *Bacillus* has the added advantage as a DFM of producing spores that are resistant to low pH, bile salts, and other harsh conditions of the gastric environment (16–19). In poultry, multiple strains of *B. subtilis* have been used as DFMs to promote growth, immunity, and overall gut health (11, 12). In humans, both observational studies and randomized controlled trials have documented the health-promoting effects of probiotic *Bacillus* spp., particularly when used to treat intestinal disorders (20).

Our prior report demonstrated that dietary supplementation of healthy broiler chickens with *B. subtilis* strain 1781 as a DFM increased body weight gains, compared with chickens fed an unsupplemented diet (21). Further, following infection with *Eimeria maxima*, oral supplementation of chickens with *B. subtilis* reduced the clinical signs of experimental avian coccidiosis, compared with unsupplemented and infected controls (22). Independently, we also reported that the AGPs, virginiamycin and bacitracin methylene disalicylate, altered the chicken intestinal metabolome and speculated that their growth enhancing effects were due to the selection of gut microbial species capable of producing metabolites that promoted more efficient energy utilization (23). Therefore, the current study was undertaken to characterize the metabolic alterations in the chicken gut following dietary supplementation with *B. subtilis* DFMs with the goal of identifying potential chemical compounds that might be directly used to improve poultry growth performance without the use of AGPs.

## Methods

### Animals

Eighty-four, day-old, male Ross 708 broiler chickens (Longenecker's Hatchery, Elizabethtown, PA) were randomly housed in starter brooder cages (Petersime, Zulte, Belgium) and provided with starter feed.

### Experimental Design, Growth Performance, and Intestinal Metabolomics Analysis

At 14 days of age, chickens were allocated to 1 of 3 dietary treatments in a randomized complete block design. The dietary treatments included a basal diet (Table 1), or a basal diet supplemented with 1.5 × 10^5^ CFU/g feed of *B. subtilis* 1781 or *B. subtilis* 747 (Arm & Hammer Co., Inc., Waukesha, WI). The dose of *B. subtilis* was chosen based on the previous study of Lee et al. (24). Each treatment group contained 4 cages with 7 chickens/cage. Cage dimensions were 0.65 × 0.75 m for a total of 0.4875 m^2^, resulting in 14.4 chickens/m^2^. All chickens were housed in the same room and provided *ad-libitum* access to water and feed throughout the study. Feed additions were weighed and recorded daily, and feeders were shaken once per day. The chickens and feed were weighed at 14 and 21 days of age for computation of growth performance. Dead chickens were removed and weighed to calculate mortality and adjust growth performance data. At 21 days of age, 8 chickens/group were euthanized by cervical dislocation and the intestinal ileum harvested. Intestinal ileal contents were collected aseptically by gently finger-stripping the ileal segment, immediately placed on dry ice, and stored at −80°C. Global metabolomic profiling of the intestinal contents was performed by mass spectrometry (MS) (Metabolon, Durham, NC) as described (23). Raw data was extracted and processed using the DiscoveryHD4 global metabolomics platform. Compounds were identified by comparison to library entries of purified standards or recurrent unknown entities based on retention index, accurate mass match to the library ±10 ppm, and MS/MS forward and reverse scores between experimental data and authentic standards. MS/MS scores were based on comparison of the ions present in the experimental spectrum to the ions present in the library spectrum.

**Table 1:** Composition of the basal diet.

**Ingredient**	**%**
Corn	69.01
Soybean meal	23.99
Soybean oil	2.75
Dicalcium phosphate	2.0
Calcium carbonate	1.4
Salt	0.35
Poultry vitamin mix^a^	0.2
Poultry mineral mix^b^	0.15
DL-Methionine	0.1
Choline chloride (60%)	0.05
Total	100.0
**Calculated Nutrient Composition**	**%**
Crude protein	18.0
Calcium carbonate	1.19
Available phosphorus	0.54
Lysine	1.0
Methionine	0.42
Cysteine + Methionine	0.65
True metabolizable energy, Mcal/kg	3.59

a The vitamin mixture provided the following nutrients per kg of diet: vitamin A, 2,000 IU; vitamin D_3_, 22 IU; vitamin E, 16 mg; vitamin K, 0.1 mg; thiamin, 3.4 mg; riboflavin, 1.8 mg; vitamin B_6_, 6.4 mg; vitamin B_12_, 0.013 mg; biotin, 0.17 mg pantothenic acid, 8.7 mg; folic acid, 0.8 mg; niacin, 23.8 mg.

b *The mineral mixture provided the following nutrients per kg of diet: Fe, 0.4 mg; Zn, 0.2 mg; Mn, 0.18 mg; Co, 0.0013 mg; Cu, 0.021 mg; Se, 0.0002 mg*.

### Statistical Analysis

Each cage was considered the experimental unit. The type of experimental diet was considered the treatment factor, and each cage was considered as a blocking factor. Data were analyzed using a mixed model methodology (PROC MIXED, SAS Institute, Cary NC). For growth performance, mean ± SEM values were calculated for initial body weight (IBW) at 14 days, final body weight (FBW) at 21 days, body weight gain (BWG) between 14 and 21 days, feed intake (FI) between 14 and 21 days, and feed efficiency (FE = BWG/FI) between 14 and 21 days. Differences between means were compared using the 2-tailed Student's *t*-test with *p* ≤ 0.05 considered significantly different. All data analysis were conducted with the same way of Gadde et al. (23). Briefly, ANOVA was used to identify the biochemical changes among the 3 dietary groups following median scaling, log transformation, and imputation of missing values. Array Studio software (OmicSoft, Cary, NC) was used for Standard statistical analyses of log-transformed data. For analyses that were not standard in Array Studio, the programs R (R Foundation for Statistical Computing, Vienna, Austria) or JMP (SAS Institute) were used. Changes in biochemical levels with *p* ≤ 0.05 were considered statistically significant. To measure biochemical importance, Random Forest Analysis (RFA) was performed by computing the Mean Decrease Accuracy (MDA).

## Results

### *B. subtilis* DFMs Increase Chicken Growth Performance

Chickens were fed from 14 to 21 days post-hatch with a basal diet, or a basal diet supplemented with 1.5 × 10^5^ colony forming units (CFU)/g feed of *B. subtilis* strain 1781 or *B. subtilis* strain 787. Body weights at 14 and 21 days of age, as well as feed intake and feed efficiency between 14 and 21 days, were identical between the 3 groups (Table 2). However, body weight gains between 14 and 21 days were greater for chickens fed with either of the *Bacillus-*supplemented diets, compared with unsupplemented controls.

**Table 2:** Growth performance of chickens fed an unsupplemented control diet or a diet containing *B. subtilis* strain 1781 or *B. subtilis* strain 747.

	**Control**	***B. subtilis* 1781**	***B. subtilis* 747**
IBW, g	515 ± 5.2	526 ± 7.3	516 ± 7.3
FBW, g	866 ± 7.4	903 ± 10.4	898 ± 10.4
BWG, g	354 ± 5.6^a^	374 ± 7.9^b^	381 ± 7.9^b^
FI, g	616 ± 20.7	589 ± 29.3	591 ± 29.3
FE	0.575 ± 0.021	0.635 ± 0.030	0.645 ± 0.030

a, b Means in the same row with different superscripts differ (p < 0.05).

*Values are means ± SEM. Values Within the same row, values with different superscripts are significantly different (p < 0.05). IBW, initial body weight at 14 days of age; FBW, final body weight at 21 days of age; BWG, body weight gain between 14 and 21 days; FI, feed intake between 14 and 21 days; FE, feed efficiency between 14 and 21 days*.

### *B. subtilis* DFMs Alter Global Intestinal Metabolite Levels

A total of 674 biochemicals were identified in the intestinal contents of chickens fed an unsupplemented, control diet, or a diet supplemented with *B. subtilis* 1781 or *B. subtilis* 747. In the *B. subtilis* 1781 vs. control groups, the levels of 209 metabolites were increased and 461 were decreased. Of these, 25 of the increased and 58 of the decreased compounds were statistically significant (*p* < 0.05) In the *B. subtilis* 747 vs. control groups, 265 metabolites were increased and 402 were decreased. Of these, 12 of the increased and 38 of the decreased compounds were statistically significant (*p* < 0.05). In the *B. subtilis* 747 vs. *B. subtilis* 747 groups, 383 metabolites were increased and 279 were decreased. Of these, 18 of the increased and 4 of the decreased compounds were statistically significant (*p* < 0.05).

### Intestinal Metabolite Signatures and Biochemical Importance Analyses

A random forest analysis (RFA) was performed to identify metabolite signatures and the biochemical importance of the 30 most significantly altered metabolites for distinguishing the *B. subtilis* 1781 vs. control, *B. subtilis* 747 vs. control, and *B. subtilis* 1781 vs. *B. subtilis* 747 groups (Table 3). RFA of the *B. subtilis* 1781 vs. control groups gave a predictive accuracy of 87.5%, while that of the *B. subtilis* 747 vs. control groups was 62.5%, suggesting that these metabolites are candidate biomarkers for distinguishing between each of the 2 treatment groups and the control group. RFA of the *B. subtilis* 1781 vs. *B. subtilis* 747 groups gave a predictive accuracy of 75.0%. Compared with the control group, all 8 intestinal samples analyzed from the *B. subtilis* 1781 group, and 5 samples from the *B. subtilis* 747 group, were predicted to belong to their respective group. Three samples from *B. subtilis* 747 group were predicted to belong to the control group. Of 8 control group samples, 2 were predicted to belong to the *B. subtilis* 1781 group and 3 were predicted to belong to the *B. subtilis* 747 group. When compared between the 2 *B. subtilis* DFM groups, 8 of the *B. subtilis* 1781 samples, and 4 of the *B. subtilis* 747 samples, were predicted to belong to their respective group.

**Table 3:** Random forest analysis of the altered biochemicals distinguishing between the *B. subtilis* 1781 vs. control, *B. subtilis* 747 vs. control, and *B. subtilis* 1781 vs. *B. subtilis* 747 groups based on 8 independent samples.

		**Predicted Group**		**Class Error**
		***B. subtilis* 1781**	**Control**	
Actual group	*B. subtilis* 1781	8	0	0.0%
	Control	2	6	25.0%
Predictive Accuracy = 87.5%				
		***B. subtilis*** **747**	**Control**	
Actual group	*B. subtilis* 747	5	3	37.5%
	Control	3	5	37.5%
Predictive Accuracy = 62.5%				
		***B. subtilis*** **1781**	***B. subtilis*** **747**	
Actual group	*B. subtilis* 1781	8	0	0.0%
	*B. subtilis* 747	4	4	50.0%

*Predictive Accuracy = 75.0%*.

### Specific Intestinal Metabolites Altered Following Dietary *B. subtilis* Supplementation

**Figure 1:**
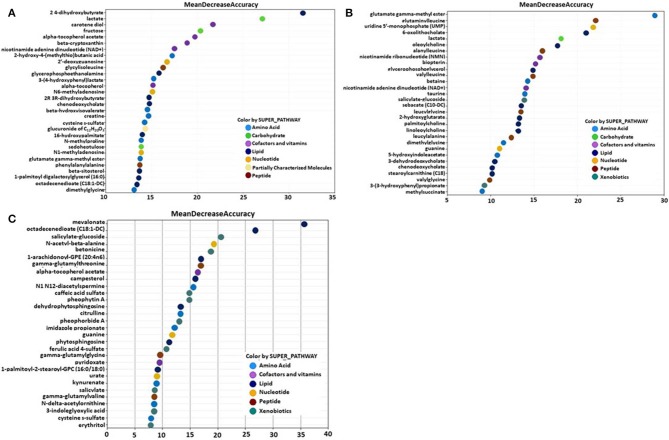
Random forest plots of the top 30 biochemicals whose levels were altered in the **(A)** *B. subtilis* 1781 vs. control, **(B)** *B. subtilis* 747 vs. control, and **(C)** *B. subtilis* 1781 vs. *B. subtilis* 747 groups. Biochemicals are listed from bottom to top in increasing order of importance for contributing to the biochemical signatures separating the respective treatment groups, and are plotted in color-coded symbols according to chemical classification.

**Figure 2:**
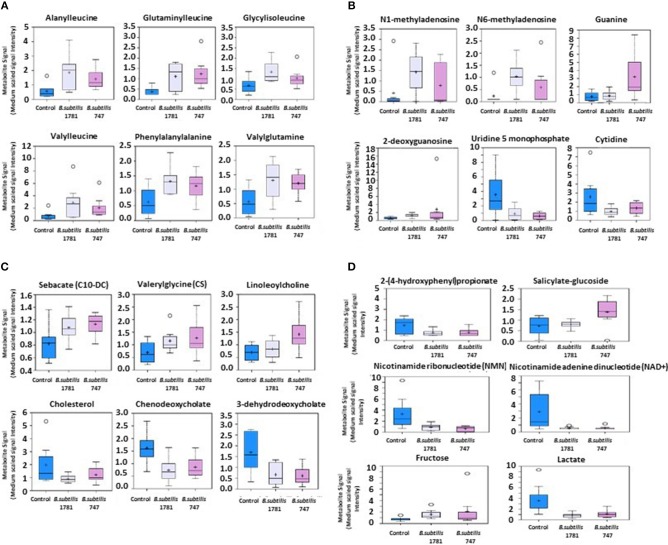
Box-and-whisker plots of the levels of **(A)** amino acids, **(B)** nucleotides, **(C)** fatty acids, and **(D)** others, including xenobiotics, vitamins, cofactors, and carbohydrates in the intestine of chickens fed an unsupplemented control diet (blue), or a diet supplemented with *B. subtilis* 1781 (gray) or *B. subtilis* 747 (pink). The boxes represent the interquartile range (IQR) defined by the 25th and 75th percentiles. The horizontal line represents the median value. The cross represents the mean value. The upper whisker represents Q3 + (1.5 × IQR), while the lower whisker represents Q1 – (1.5 × IQR). Circles represent outliers.

Metabolites of amino acids (26.7%), lipids (26.7%), vitamins and cofactors (16.7%), and nucleosides (10.3%) accounted for the majority of biochemicals classified as the most important for distinguishing between the *B. subtilis* 1781 vs. control groups (Figure 1A). Metabolites of lipids (33.0%), amino acids (20.0%), and peptides (20.0%) accounted for the majority of biochemicals for distinguishing between the *B. subtilis* 747 vs. control groups (Figure 1B). Metabolites of carbohydrates (30.0%), amino acids (20.0%), and lipid (20.0%) accounted for the majority of biochemicals for distinguishing between the *B. subtilis* 1781 vs. *B. subtilis* 747 groups (Figure 1C). Among the amino acid metabolites most highly elevated in the *B. subtilis* 1781 vs. control and *B. subtilis* 747 vs. control groups were leucine-containing dipeptides. The levels of alanylleucine (Ala-Leu), glutaminylleucine (Gln-Leu), valylleucine (Val-Leu), and glycylisoleucine (Gly-Ile) were increased 3.28-, 3.01-, 3.98-, and 1.98-fold, respectively, in the intestinal contents of chicken fed the *B. subtilis* 1781 diet, compared with unsupplemented controls (Figure 2A). These same dipeptides were increased 2.49-, 3.34-, 1.53- and 2.82-fold in *B. subtilis* 747-treated chickens, compared with controls. The alanine-associated dipeptide phenylalanylalanine (Phe-Ala), and the glutamine-associated dipeptide valylglutamine (Val-Gln) were increased 2.12- and 2.31-fold in the *B. subtilis* 1781 vs. control groups, and 1.87- and 2.14-fold in the *B. subtilis* 747 vs. control groups. Biochemicals associated with purine metabolism that were increased in the *B. subtilis* 1781- or *B. subtilis* 747-supplemented diets vs. controls included *N*1-methyladenosine (3.45-, and 1.89-fold, respectively), *N*6-methyladenosine (4.43-, 2.51-fold), guanine (1.10-, 4.12-fold), and 2-deoxyguanosine (2.00-, 4.42-fold) (Figure 2B). Biochemicals associated with pyrimidine metabolism that were decreased in the *B. subtilis* 1781 or *B. subtilis* 747-supplemented diets vs. controls included uridine-5′-monophosphate (0.26-, and 0.17-fold) and cytidine (0.37-, and 0.52-fold) (Figure 2B). Fatty acids and their metabolites also contributed to the biochemical signatures that distinguished chickens given the *B. subtilis*-supplemented diets from unsupplemented controls. For example, sebacate (C10-DC), valerylglycine, and linoleoylcholine were increased 1.34-, 1.74, and 1.62-fold in the *B. subtilis* 747 diet vs. controls (Figure 2C). By contrast, sterols and bile acids were decreased in both *Bacillus subtilis* vs. control groups. Cholesterol, chenodeoxycholate, and 3-dehydrodeoxycholate were decreased 0.54-, 0.50-, and 0.38-fold in the *B. subtilis* 1781-supplemented diet vs. controls (Figure 2C). Related to benzoate metabolism, the levels of 2-(4-hydroxyphenyl)propionate were decreased in both *B. subtilis* groups vs. control group (~0.53-fold), whereas the levels of salicylate-glucoside were increased in the *B. subtilis* 747 vs. control groups (1.92-fold) (Figure 2D). Related to nicotinamide metabolism, the levels of nicotinamide ribonucleotide (NMN) and nicotinamide adenine dinucleotide (NAD+) were reduced <80% in both *B. subtilis*-supplemented chickens, compared with controls. Finally, related to carbohydrate metabolism, fructose levels were elevated in the *B. subtilis* 1781 vs. control (2.01-fold) and *B. subtilis* 747 vs. control (2.64-fold) groups, while lactate levels were decreased in both *B. subtilis* groups vs. control group (0.27-fold) (Figure 2D).

## Discussion

We previously reported that dietary supplementation of broiler chickens with *B. subtilis*-based DFMs increased body weight gains, compared with unsupplemented controls (21). Further, chickens fed a *B. subtilis*-containing diet had increased intestinal villus height and crypt depth, augmented mitogen- and antigen-induced spleen cell proliferation, greater macrophage phagocytosis of *Salmonella* bacteria, decreased serum levels of α-1-acid glycoprotein and nitric oxide, altered expression of cytokine genes (interferon-γ, interleukin-1β, CXCL2) in intestinal lymphocytes, and a modified CD4^+^/CD8^+^ ratio of peripheral blood lymphocytes (24–28). The current study extends these prior results to now demonstrate that dietary supplementation with *B. subtilis* 1781 or *B. subtilis* 787 alters the chicken intestinal metabolome.

Supplementing these studies, other investigators have documented the growth promoting effects of *Bacillus*-based DFMs in poultry. Opalinski et al. (29) reported reduced feed conversion ratio (FCR), a measure of feed input divided by body weight gain, in chickens fed a diet supplemented with *B. subtilis*, compared with unsupplemented controls, although final weight gains were unaffected. Aliakbarpour et al. (30) confirmed that dietary *B. subtilis* decreased FCR, and increased weight gains and MUC2 mucin gene expression in intestinal goblet cells, compared with controls. Jeong et al. (31) replicated the effects of *B. subtilis* DFMs on chicken FCR and weight gains, and also demonstrated increased *Lactobacillus* spp. and reduced *Escherichia coli* levels in the intestinal contents of DFM-supplemented birds, compared with controls. Increased growth performance, nutrient utilization, and intestinal villus/crypt morphometry, as well as altered composition of gut microbiota, also were observed in chickens orally administered with *Bacillus* DFMs, compared with controls (32–35).

In addition to their effects on healthy animals, *Bacillus* DFMs also have been shown to ameliorate the deleterious outcomes associated with enteric bacterial infections of poultry. *B. subtilis* dietary supplementation reversed the effects of *Clostridium perfringen*s infection on decreased growth performance and alterations in the intestinal microbiota, compared with unsupplemented controls (24). In a *C. perfringens*/*Eimeria maxima* coinfection model of avian necrotic enteritis, feeding of *B. subtilis* reduced gut pathology and animal mortality, and restored alterations in the intestinal microbiome, compared with controls (22). Other studies have confirmed the ability of *Bacillus* DFMs to reduce *C. perfringens* colonization of the intestinal mucosa and improve animal growth performance in avian necrotic enteritis (36–41). In general, while the beneficial effect of *Bacillus* DFMs on body weight gains in healthy, uninfected chickens is generally moderate (<10%), a more pronounced outcome of the dietary supplements has been observed when weight loss is aggravated as a consequence of pathogenic infection (42).

Given the reported effects of DFMs on modulating the chicken gut microbiome (10–12), and the ability of intestinal bacteria to synthesize vitamins and nutrients beneficial to the host (43), it is somewhat surprising that few reports have examined the ability of DFMs to alter the avian metabolome (35, 44). Cao et al. (35) reported that dietary supplementation of chickens with *B. amyloliquefaciens* altered the levels of gut metabolites related to amino acid and glyceride metabolism. More specifically, increased levels of 4-aminobutyric acid, gentiobiose, quinic acid, 3,7,12-trihydroxycoprostane, N-ethylglycine, glycine, N-acetyl-D-galactosamine, and 5-hydroxyindole-3-acetic acid, and reduced the levels of diglycerol and N-acetyl-β-D-mannosamine, were seen in the DFM-supplemented chickens, compared with unsupplemented controls. In another study, Wang et al. (44) observed that chickens fed a diet containing the yeast *Kluyveromyces marxianus* had altered serum levels of 60 biochemical metabolites (39 increased, 21 decreased), compared with controls. Biochemicals involved in carbohydrate and amino acid, primarily glutamate and glutamine, metabolism were identified as the most relevant.

In humans, probiotics have been used to treat a variety of diseases and disorders, and in some cases, metabolic profiling has begun to unravel their mechanisms of action. For example, in a double-blind, randomized, placebo-controlled clinical trial of a fermented milk probiotic provided to patients with irritable bowel syndrome, decreased selected clinical symptoms was correlated with increased serum glucose and tyrosine levels (45). In women with mastitis, consumption of a *Lactobacillus* probiotic decreased staphylococcal/streptococcal bacterial load in breast milk and breast pain, while increasing urinary levels of creatinine, hippuric acid (a glycine conjugate of benzoic acid), and trimethylamine-N-oxide (a choline metabolite) (46). Oral administration of a *Bifidobacterium animalis* probiotic to patients with atopic dermatitis improved dermatology-specific quality-of-life scores, while increasing fecal kynurenic acid and decreasing 3-hydroxyproprionic acid levels, compared with placebo controls (47). In a study of chronic kidney disease patients orally administered with a probiotic containing *Streptococcus thermophilus, Lactobacillus acidophilus*, and *Bifidobacterium longum*, serum levels of metabolites related to carbohydrate, choline, and energy metabolism differentiated individuals with increased vs. decreased blood urea nitrogen levels (48). *Per os* administration of a probiotic mixture containing 4 strains of lactobacilli, 3 strains of bifidobacteria, and 1 strain of *Streptococcus thermophilus* to infants with colic reduced clinical symptoms, while increasing fecal levels of alanine, leucine, isoleucine, acetate, and pyruvate and decreasing the levels of uracil and propylene glycol, compared with placebo controls (49). Finally, a similar relationship between reduction of disease scores and alterations in metabolic profiles induced by administration of bacterial probiotics has been reported in animal models of human diseases, including fecal metabolites in mice with chemical-induced colits (50) and serum metabolites in rats with depression-related behavioral changes (51).

The alteration of metabolites observed in the current study suggest that these small molecules (gut metabolites) might play a role in the recognition of pathogen-associated molecular patterns (PAMPs) through influencing the host immune response to maintain homeostasis against intestinal diseases and inflammation. In this way, these responses may ultimately lead to improved growth performance (52–55). Short-chain fatty acids (SCFAs), such as acetate, n-propionate, and n-butyrate, are known to play a role in the recognition of PAMPs (52), however, alternations in SCFA levels in the gut were not found in the current study. SCFAs can be sensed by G-protein coupled receptors (GPRs) which are expressed on many cells, such as epithelial cells, neutrophils, and macrophages (52). GPR pathways are associated with cytokines and tight junction protein expression (56, 57). Lipid metabolites in the current study may also be sensed by GPRs, which implies that lipid metabolites can affect the regulation of immunity or inflammation in the gut.

In summary, to the best of our knowledge, this is the first report to demonstrate that dietary supplementation with *B. subtilis* has profound effects on the levels of a wide variety of chemical metabolites in the chicken gut, particularly those related to amino acids, nucleosides, fatty acids, and carbohydrates. Compared with unsupplemented controls, these altered metabolite levels provide a biochemical signature unique to each *B. subtilis* supplementation group. Our result suggest that altered metabolites can be used to maintain gut homeostasis within epithelial or immune cells, which might account for their affect on overall gut health as well as chicken growth. Through future *in vitro* and/or *in vivo* studies, identification of the altered metabolites that confer properties of AGPs would suggest their potential use as antibiotic alternatives.

## Data Availability Statement

All datasets generated for this study are included in the article/supplementary material.

## Ethics Statement

The animal study was reviewed and approved by the Beltsville Agricultural Research Center Institutional Animal Care and Use Committee and performed in accordance with the guidelines and regulations.

## Author Contributions

IP and HL designed the research and conducted research. IP, EL, and HL analyzed data. IP, NZ, AS, TR, EL, and HL had responsibility for content. All authors read and approved the final manuscript.

### Conflict of Interest

NZ, AS, and TR were employed by the Arm & Hammer Animal and Food Production. The remaining authors declare that the research was conducted in the absence of any commercial or financial relationships that could be construed as a potential conflict of interest.
